# Supplementary material for: Gene Expression Profiling as a Novel Diagnostic Tool for Neurodegenerative Disorders
Source: Int J Mol Sci. 2023 Mar 17;24(6):5746. doi: 10.3390/ijms24065746 (PMC10057696; doi:10.3390/ijms24065746)
Supplement: Supplementary file 1 [file ijms-24-05746-s001.zip › ijms-2237209-supplementary.pdf]

Supplementary Table S1. Diagnosis and Demographics of Patients with Different *PSEN*, *PICALM*, *ABCA7* and *SNCA* genotypes.

GENOTYPE	DIAGNOSIS	GENOTYPE	DIAGNOSIS	GENOTYPE	DIAGNOSIS	GENOTYPE	DIAGNOSIS
PSEN TT *(n=7, 3F and 4M; age: 70.71*±3.99)	NCD mixed dementia	***PICALM CC*** *(n=7, 3F and 4M, age: 74.41*±5.47)	NCD mixed dementia	***ABCA7 TT*** *(n=10, 6F and 4M; age: 75.18*±5.13)	NCD mixed dementia	***SNCA GG*** *(n=4, 2F and 2M, age: 68*±7.79)	NCD mixed dementia
	PD		NCD		NCD mixed dementia		Ataxia
	NCD mixed dementia		NCD mixed dementia		NCD mixed dementia		PD
	NCD		NCD mixed dementia		NCD mixed dementia		PD
	NCD-AD		NCD		Encephalopathy	***SNCA GA*** *(n=5, 2F and 3M, age: 68.8*±6.98)	NCD mixed dementia
	Encephalopathy		NCD		NCD-AD		PD
	PD		Vascular headache		NCD-AD		PD
PSEN GT *(n=7, 4F and 3M; age: 68.57*±5.74)	PD	***PICALM CT*** *(n=7, 3F and 4M, age: 72*±3.79)	NCD mixed dementia		NCD-AD		NCD
	NCD mixed dementia		NCD mixed dementia		Vascular headache		PD
	PD		NCD mixed dementia		Encephalopathy	***SNCA AA*** *(n=7, 3F and 4M, age: 70.86*±3.72)	PD
	PD		NCD	***ABCA7 TG*** *(n=3, 3F and 3M; age: 74.56*±5.28)	NCD mixed dementia		PD
	NCD mixed dementia		NCD		NCD mixed dementia		PD
	Vascular headache		NCD mixed dementia		NCD mixed dementia		NCD
	Encephalopathy		NCD		NCD mixed dementia		Encephalopathy
PSEN GG *(n=6, 3F and 3M; age: 72.86*±2.27)	NCD mixed dementia		NCD-AD		Encephalopathy		Vascular headache
	NCD	***PICALM TT*** *(n=5, 3F and 2M, age: 74.29*±6.58)	NCD		NCD-AD		Encephalopathy
	NCD mixed dementia		NCD-AD	***ABCA7 GG*** *(n=7, 3F and 4M; age: 67.8*±7,63)	NCD mixed dementia		
	PD		Vascular headache		NCD mixed dementia		
	NCD		NCD		NCD mixed dementia		
	Encephalopathy		NCD		NCD mixed dementia		
					Encephalopathy		
					Cephalea		
					Encephalopathy		

AD, Alzheimer´s disease; Genotypes: AA, CC, CT, GA, GG, GT, TG, TT; NCD, neurocognitive disorder; PD, Parkinson´s disease; Sex: M, male; F, female.
